# Distinct patterns of spontaneous brain activity in mild cognitive impairment patients stratified by cerebrospinal fluid biomarkers

**DOI:** 10.3389/fnagi.2026.1724058

**Published:** 2026-03-27

**Authors:** Wenzhang Qi, Changbao Zhang, Yuchen Feng, Darui Zheng, Yiming Ruan, Qianqian Yuan, Ke Dai, Chen Xue

**Affiliations:** 1 Department of Radiology, The Affiliated Brain Hospital of Nanjing Medical University, Nanjing, China; 2 Department of Radiology, Shanghai Tenth People’s Hospital, Tongji University School of Medicine, Shanghai, China; 3 School of Clinical and Basic Medicine, Institute of Basic Medical Sciences, Shandong First Medical University and Shandong Academy of Medical Sciences, Jinan, China

**Keywords:** amplitude of low-frequency fluctuation, amyloid-beta, functional magnetic resonance imaging, mild cognitive impairment, tau protein

## Abstract

**Background:**

This study aimed to explore the alterations in the Amplitude of Low-Frequency Fluctuation (ALFF) among mild cognitive impairment (MCI) patients with varying cerebrospinal fluid (CSF) biomarker levels, including abnormal Amyloid-beta (Aβ42) and phosphorylated tau protein (p-tau) (A + T +), abnormal Aβ42 and normal p-tau (A + T-), and normal Aβ42 and p-tau (A-T-), and to investigate their longitudinal changes.

**Methods:**

A total of 134 MCI patients were enrolled in this study and stratified into three groups, including 54 A-T- group, 28 A + T- group, and 52 A + T + group based on CSF Aβ and p-tau levels. The baseline ALFF values derived from blood oxygen level-dependent signals were employed to quantify spontaneous brain activity patterns. Comparisons were made among the three groups regarding ALFF differences, and their correlations with cognitive functions and CSF biomarkers were investigated. Additionally, a subset of participants with 2-year follow-up data underwent a mixed-effects model analysis to examine group-by-time interactions in ALFF.

**Results:**

At baseline, compared to the A-T- group, the A + T- group showed decreased ALFF in the bilateral cerebellar posterior lobe (CPL) and increased ALFF in the bilateral middle frontal gyrus (MFG), while the A + T + group displayed decreased ALFF in the bilateral CPL. In contrast to the A + T- group, the A + T + group exhibited decreased ALFF in the bilateral MFG. Additionally, there was a significant negative correlation between the bilateral MFG and p-tau levels, while the left MFG was positively correlated with RAVLT-learning performance, and the right MFGs was positively correlated with Aβ levels. Longitudinally, a significant group-by-time interaction was observed in the right inferior temporal gyrus (ITG), where A + T + patients exhibited a more pronounced ALFF decline over 2 years compared to A + T- patients.

**Conclusion:**

This study highlights distinct and evolving patterns of brain activity associated with Aβ and tau pathology in MCI. The combination of cross-sectional and longitudinal analyses reveals that tau pathology may drive accelerated functional decline in specific brain regions, particularly the right ITG, offering insights into the progression of AD-spectrum disorders.

## Introduction

Mild Cognitive Impairment (MCI) is often regarded as a precursor to Alzheimer’s Disease (AD), sitting between normal aging and AD (Jessen et al., 2014). However, MCI is actually a heterogeneous disorder with a variable progression; not all MCI patients will develop AD (Petersen et al., 2014). Furthermore, the clinical diagnosis of MCI primarily relies on clinical scales and symptomatic assessments, which can be subjective and may not accurately reflect the patient’s true condition. To more accurately diagnose AD and its precursor stages, recent AD research diagnostic guidelines have proposed the use of Aβ and tau protein biomarkers for diagnosis (Dubois et al., 2014; Jack et al., 2016; Jack et al., 2018). These guidelines introduce the A/T/N system, which assesses AD pathological status by measuring amyloid-beta (Aβ, A), tau protein (T), and biomarkers of neurodegeneration or neuronal injury (N) (Jack et al., 2016). The ATN classification system further hypothesizes that the combination of abnormal Aβ and tau biomarkers most accurately reflects the presence of AD pathology, which is significant for understanding the pathological mechanisms and diagnosis of AD (Kim et al., 2021; Zhang et al., 2021).

Over the past decade, resting-state functional magnetic resonance imaging (rs-fMRI) technology has been widely used in neuroscientific research due to its non-invasiveness, ease of use, and reproducibility (Xue et al., 2020; Xue et al., 2021). As a commonly used measurement, Amplitude of Low-Frequency Fluctuation (ALFF) quantifies local neural activity in low-frequency fluctuations of blood oxygen level-dependent signals during resting states (Zuo et al., 2010; Jia et al., 2020). ALFF has high test-retest reliability and does not require prior hypotheses about specific brain regions being studied during the calculation process (Chen et al., 2018). Recent studies found similar changes in ALFF in AD and preclinical AD patients, which were related to the neurotransmitter system (Tang et al., 2024). However, there was still a lack of in-depth research on spontaneous brain activity changes in MCI patients with different Aβ and tau protein levels. In addition, most prior studies investigating brain functional alterations in MCI were cross-sectional in nature, which limits the ability to capture the dynamic progression of neurodegenerative changes. Longitudinal assessments are essential to reveal how spontaneous brain activity evolves with disease advancement and to distinguish transient compensatory responses from sustained functional decline.

Therefore, the primary objective of this study was to explore the effects of different levels of Aβ and tau pathology on brain function. Based on the ATN classification system, we employed ALFF analysis to analyze brain activity changes in MCI patients with different cerebrospinal fluid (CSF) pathology protein levels. Furthermore, correlation analysis was conducted to assess the relationship between altered ALFF values, cognitive function and CSF indicators, including Aβ and phosphorylated tau protein (p-tau). More importantly, this study further incorporated a 2-year follow-up analysis using a mixed-effects model to examine longitudinal changes in ALFF and their association with CSF biomarker-defined MCI subtypes. We hypothesized that different levels of Aβ and tau pathology would not only result in distinct patterns of spontaneous brain activity and cognitive performance at baseline, but also lead to divergent trajectories of functional brain changes over time. These alterations may reflect the dynamic progression of neurodegeneration and help elucidate the underlying mechanisms of MCI within the Alzheimer’s disease spectrum.

## Materials and methods

### Subjects

The data utilized in this study were all sourced from the Alzheimer’s Disease Neuroimaging Initiative (ADNI) database.^1^ The primary objective of ADNI is to validate AD biomarkers and investigate their use in combination with clinical and neuropsychological assessments to evaluate the clinical progression of AD individuals with MCI or early AD.

For this study, we retrieved all 226 MCI subjects with CSF data from the ADNI2 and ADNI3 databases. After excluding those with excessive head movement, 212 patients remained. According to the previous study, a cutoff value of < 977 pg/ml was applied to determine CSF Aβ_42_ abnormality, and > 24 pg/ml was used for CSF p-tau abnormality (Hansson et al., 2018; Vromen et al., 2023). Based on these criteria, the MCI patients were categorized including abnormal Aβ_42_ and p-tau (A + T +), abnormal Aβ_42_ and normal p-tau (A + T-), normal Aβ_42_ and abnormal p-tau (A-T +), and normal Aβ_42_ and p-tau (A-T-). The A-T + group was excluded due to their classification outside the AD spectrum according to the A/T/N framework (Vromen et al., 2023). Ultimately, the present study included 54 A-T- participants, 28 A + T- participants, and 52 A + T + participants.

For longitudinal analysis, subjects who completed both baseline and 2-year follow-up MRI scans and cognitive assessments were considered. After applying quality control procedures for motion and data completeness, 69 individuals remained for longitudinal analysis, consisting of 22 A- T-, 16 A + T-, and 31 A + T + participants.

### Ethics approval and consent to participate

Ethical approval for the ADNI study was granted by the institutional review committees of all participating institutions. Written informed consent was obtained from participants or their authorized representatives, and additional details can be accessed on the ADNI website.^2^

### Cognitive function

For assessing cognitive function, we conducted comparisons between groups using the composite episodic memory (EM) score and the composite executive function (EF) score. EM was evaluated by the composite score of the Rey Auditory Verbal Learning Test, the Alzheimer Disease Assessment Scale-Cognitive, Logical Memory, and MMSE. EF was assessed by the composite score of Category Fluency, WAIS-R Digit Symbol, Trails A and B, Digit Span Backwards, and clock drawing. For all composite measures, higher values indicated better cognitive performance. All neurocognitive assessments are available on the ADNI website.^3^

### Pathological sample acquisition

The CSF samples were collected per the Alzheimer’s Association Flow Chart for CSF biomarkers. The INNO-BIAALZBio3 immunoassay kit was used to determine CSF levels of Aβ, total tau protein (t-tau), and p-tau.

### MRI data acquisition

Detailed scanning information can be obtained from http://adni.loni.usc.edu/wp-content/uploads/2010/05/ADNI2 MRI Training-Manual-FINAL.pdf and http://adni.loni.usc.edu/wp-content/uploads/2017/07/ADNI3-MRI-protocols.pdf.

### Functional data preprocessing

Preprocessing of fMRI data was performed using Data Processing and Analysis for Brain Imaging (DPABI)^4^ software in MATLAB 2021b.^5^ The preprocessing steps were as follows: (1) discarding the first 10 functional image volumes; (2) correcting slice timing and head motion; participants with excessive head motion (cumulative translation or rotation of > 3.0 mm or 3.0°) were excluded; (3) spatially normalizing images to the Montreal Neurological Institute (MNI) echo-planar imaging template and resampled to 3 × 3 × 3 mm^3^ voxels; (4) smoothing normalized brain volumes with a Gaussian kernel of 6-mm full-width half-maximum to reduce individual variations; (5) removing nuisance variables such as 24 head motion parameters, head motion scrubbing regression, global mean signal, white matter signal, and cerebrospinal fluid signal.

### ALFF measurement

Utilizing the DPABI software, we computed the ALFF value. This involved converting the time series of each voxel into the frequency domain via the fast Fourier transform to obtain the power spectrum. The square root was calculated for each frequency within the power spectrum, and the ALFF value was determined as the average of these square roots over the frequency range of 0.01–0.08 Hz. Subsequently, the ALFF values were converted to Z scores (zALFF) to facilitate comparisons between groups.

### Statistical analysis

Statistical Package for the Social Sciences (SPSS) software, version 25.0 (IBM, Armonk, New York, NY, United States) was employed for statistical analysis. ANOVA and the chi-square test were utilized to compare demographic, neurocognitive scales, and CSF pathological protein among the three groups: A- T-, A + T-, A + T + at baseline and 2-year follow-up data. Bonferroni correction was applied for *post hoc* comparisons, and a *p* < 0.05 was considered statistically significant. In particular, CSF biomarker ratios, T-tau/Aβ_42_ (cutoff = 0.33) and P-tau/Aβ_42_ (cutoff = 0.028), were calculated, as previous studies have shown that these ratios demonstrate high agreement with PET SUVR-based classification and can predict future clinical progression in individuals with MCI (Hansson et al., 2018).

A one-way ANOVA analysis was performed within a gray matter mask to compare the differences in ALFF after controlling for the influence of age, gender, and years of education (GRF corrected, two-sided, voxel *p* < 0.005, cluster *p* < 0.05). Subsequently, the two-sample *t*-test was used for *post hoc* comparisons with the mask resulted from the ANOVA analysis with age, gender, and years of education as covariates (GRF corrected, two-sided, voxel *p* < 0.005, cluster *p* < 0.05).

Correlation analyses were performed in SPSS, investigating relationships between altered ALFF and cognitive function and CSF pathological protein, adjusting for age, sex, and years of education as covariates. Statistical significance was determined using false discovery rate (FDR) correction across all tested associations (*q* < 0.05).

To assess longitudinal changes in brain function, we further conducted a mixed-effects model analysis implemented in DPABI software. The group (A + T- vs. A + T +) was included as a between-subject factor and time (baseline vs. 2-year follow-up) as a within-subject factor, with subject treated as a random effect. Age, sex, and years of education were included as covariates. Statistical inference at the voxel level within gray matter mask was performed using GRF correction (two-sided, voxel *p* < 0.005, cluster *p* < 0.05). This analysis aimed to identify brain regions where ALFF values showed differential trajectories between the two groups over time.

To address potential variability arising from the multi-site design of the ADNI dataset, site information was incorporated as a categorical covariate in sensitivity analyses. This approach aimed to account for acquisition-related differences while preserving the primary model structure. Specific methods and related results were detailed in Supplementary material.

To evaluate potential attrition bias, baseline demographic characteristics, cognitive performance, and CSF biomarkers were compared between participants who completed the 2-year follow-up and those who did not. Detailed methods and results are provided in Supplementary material.

## Results

### Demographic and neurocognitive characteristics

As listed in Table 1, the A + T + group exhibited older than A-T- and A + T- groups. As anticipated, compared to A-T- group, A + T + group showed lower scores in immediate and learning part of Rey Auditory Verbal Learning Test (RAVLT), Logical Memory Test (LMT), EM, and Aβ_42_ and higher scores in t-tau, p-tau, T-tau/Aβ_42_, and P-tau/Aβ_42_ at both baseline and 2-year follow-up while A + T- showed decreased LMT score and Aβ_42_ at both baseline and 2-year follow-up. Compared to A + T- group, A + T + group showed higher scores in t-tau, p-tau, T-tau/Aβ_42_, and P-tau/Aβ_42_ at both baseline and 2-year follow-up and lower scores in Mini-mental State Examination (MMSE), RAVLT-immediate, and EM at 2-year follow-up (Bonferroni corrected, *p* < 0.05).

**TABLE 1:** Demographics and clinical measures of three groups, including A- T-, A + T-, and A + T +.

	A-T-	A + T-	A + T +	*F*-values (χ ^2^)	*p*-values
Number					
Y0	54	28	52		
Y2	22	16	31		
Age (years)	68.97(7.65)	71.68(7.30)	73.16(5.79)**	4.982	0.008^a^
Gender (F/M)	20/34	13/15	20/32	0.723	0.697
Years of education	15.91(2.64)	16.46(2.57)	16.33(2.65)	0.534	0.588
FD	0.13(0.74)	0.11(0.06)	0.13(0.08)	0.762	0.469
MMSE					
Y0	28.06(1.89)	28.18(1.74)	27.38(2.18)	2.100	0.127
Y2	27.64(2.17)	27.81(2.43)	24.90(4.66)*/*	5.296	0.007^a,c^
MoCA					
Y0	23.79(3.06)	23.04(2.52)	22.76(3.61)	1.402	0.250
Y2	23.41(3.03)	22.88(4.16)	20.00(6.11)*	3.687	0.030^a^
RAVLT-immediate					
Y0	38.43(9.42)	34.68(9.21)	33.10(8.94)*	4.620	0.012^a^
Y2	35.95(9.90)	34.44(12.24)	27.68(11.66)*	4.005	0.023^a^
RAVLT-learning					
Y0	4.91(2.24)	4.36(2.09)	3.73(2.39)*	3.559	0.031^a^
Y2	4.23(2.05)	4.56(3.12)	2.58(2.05)*/*	5.107	0.009^a,c^
RAVLT-forgetting					
Y0	4.43(3.93)	4.57(2.17)	5.10(2.51)	0.654	0.522
Y2	3.00(6.31)	4.94(2.77)	4.90(2.36)	1.623	0.205
RAVLT-prec-forgetting					
Y0	46.08(56.25)	57.15(29.42)	65.50(30.51)	2.757	0.067
Y2	35.09(86.97)	61.28(35.91)	82.73(30.99)*	4.661	0.013^a^
LDELTOTAL					
Y0	8.20(2.54)	6.21(3.06)*	5.90(3.34)***	8.809	< 0.001^a,b^
Y2	9.77(3.70)	6.56(5.48)	4.61(5.40)***	7.026	0.002^a^
EM					
Y0	0.49(5.20)	0.30(1.05)	0.05(0.65)**	5.113	0.007^a^
Y2	0.47(0.57)	0.29(0.90)	–0.45(1.04)***/*	7.921	0.001^a,c^
EF					
Y0	0.65(0.91)	0.23(1.05)	0.24(0.84)	3.310	0.040
Y2	0.54(0.89)	0.31(0.91)	–0.13(1.18)	2.845	0.065
Aβ_42_					
Y0	1484.25(255.03)	726.19(199.64)***	622.09(159.11)***	249.840	< 0.001^a,b^
Y2	1453.40(167.33)	725.77(272.44)***	569.68(238.82)***	27.180	<0.001^a,b^
T-tau					
Y0	201.89(44.35)	178.88(46.61)	383.71(130.72)***/***	73.112	< 0.001^a,c^
Y2	186.18(40.05)	198.48(52.38)	448.14(187.91)***/***	16.965	<0.001^a,c^
P-tau					
Y0	17.36(3.98)	16.30(4.68)	40.58(16.54)***/***	75.415	< 0.001^a,c^
Y2	15.52(4.19)	17.88(5.05)	46.15(21.79)***/***	16.880	<0.001^a,c^
T-tau/Aβ_42_ ratio					
Y0	0.14(0.03)	0.27(0.11)*	0.66(0.32)***/***	85.239	< 0.001^a,b,c^
Y2	0.12(0.02)	0.25(0.10)	0.83(0.54)*/*	7.412	0.004^a,c^
P-tau/Aβ_42_ ratio					
Y0	0.01(0.002)	0.02(0.01)	0.07(0.04)***/***	69.522	< 0.001^a,c^
Y2	0.01(0.002)	0.02(0.01)	0.08(0.06)*/*	6.759	0.005^a,c^

Numbers are given as means (standard deviation, SD) unless stated otherwise. FD, Framewise Displacement (Jenkinson formulation); MMSE, Mini-mental State Examination; MoCA, Montreal Cognitive Assessment; RAVLT, Rey Auditory Verbal Learning Test; LDELTOTAL, Logical Memory Test; EM, episodic memory; EF, executive function; ^a^*post hoc* analyses showed a significantly group difference between A + T + and A-T-; ^b^*post hoc* analyses showed a significantly group difference between A + T- and A-T-; ^c^*post hoc* analyses showed a significantly group difference between A + T + and A + T-; *p < 0.05; **p < 0.01; ***p < 0.001; A + T + , abnormal Aβ_42_ and p-tau; A + T-, abnormal Aβ_42_ and normal p-tau; A- T-, normal Aβ_42_ and p-tau; Aβ, Amyloid-beta protein; p-tau, phosphorylated tau protein; t-tau, total tau protein; Y0, baseline time; Y2, 2-year follow-up.

### ALFF analysis

**FIGURE 1:**
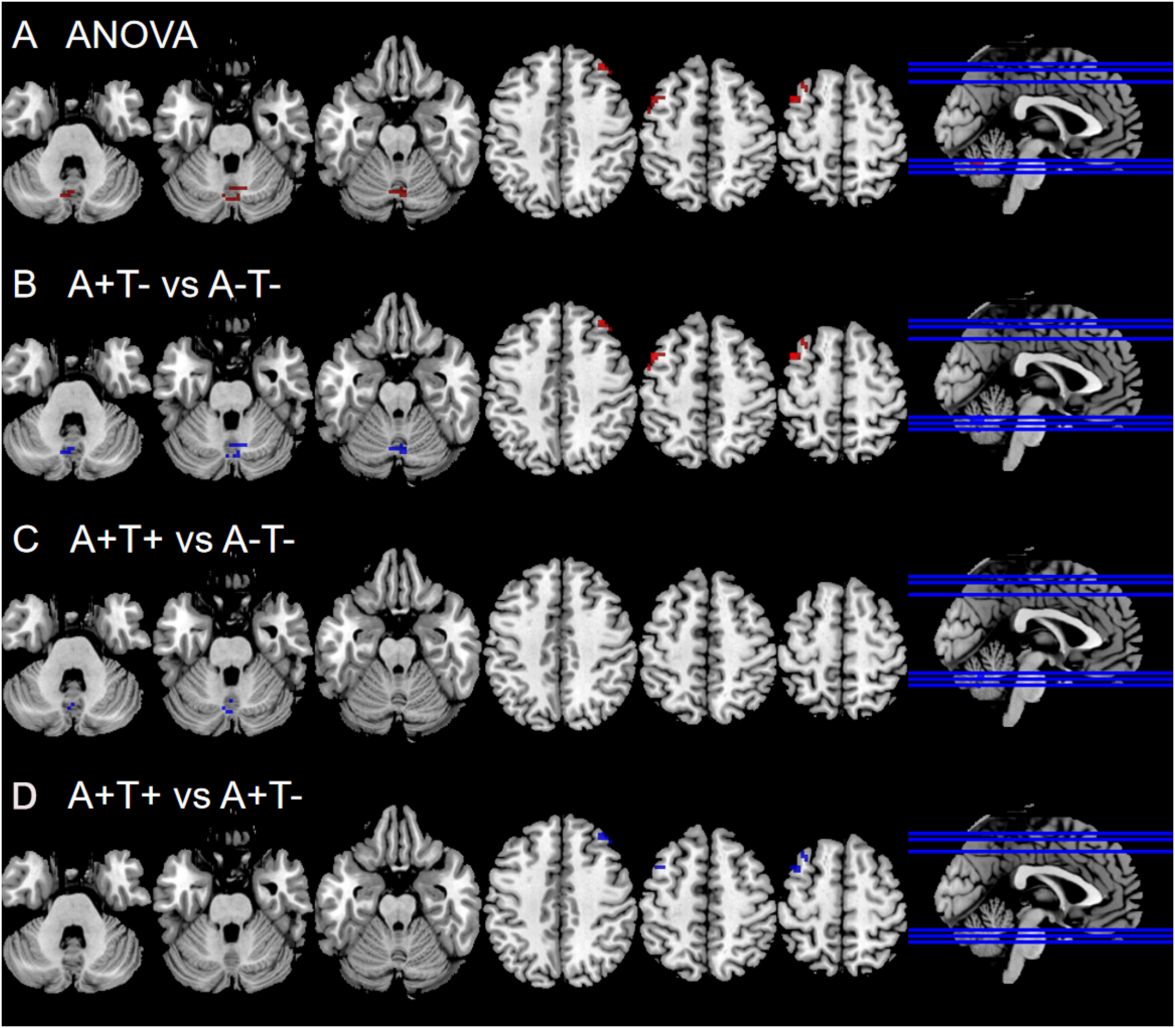
Brain regions exhibiting significant differences in ALFF. **(A)** Brain regions showing significant differences in ALFF across three groups, including A- T-, A + T-, and A + T + (GRF corrected, voxel *p* < 0.005, cluster *p* < 0.05). **(B)** Brain region showing significant differences in ALFF between A + T- and A-T- (GRF corrected, voxel *p* < 0.005, cluster *p* < 0.05). **(C)** Brain region showing significant differences in ALFF between A + T + and A-T- (GRF corrected, voxel *p* < 0.005, cluster *p* < 0.05). **(D)** Brain region showing significant differences in ALFF between A + T + and A + T- (GRF corrected, voxel *p* < 0.005, cluster *p* < 0.05). A + T + , abnormal Aβ_42_ and p-tau; A + T-, abnormal Aβ_42_ and normal p-tau; A- T-, normal Aβ_42_ and p-tau.

ANOVA showed significant alterations in ALFF values among the groups, including the bilateral cerebellum posterior lobe (CPL) and bilateral middle frontal gyrus (MFG). When compared with the A-T- group, the A + T- group showed increased ALFF values in the bilateral MFG and decreased in the bilateral CPL while the A + T + group exhibited decreased ALFF values in the bilateral CPL (GRF corrected, voxel *p* < 0.005, cluster *p* < 0.05). These results were obtained while accounting for age, sex, and years of education (Figure 1 and Table 2).

**TABLE 2:** The difference of ALFF across three groups at baseline.

Region(aal)	Peak MNI coordinate			*F/t*	Cluster number
	*x*	*y*	*z*		
ANOVA					
B cerebellum posterior lobe	9	–63	24	8.475	59
R middle frontal gyrus	33	36	45	12.1251	27
L middle frontal gyrus	–39	12	57	10.1158	44
A + T- vs. A-T-					
B cerebellum posterior lobe	9	–66	–24	–3.699	56
R middle frontal gyrus	33	39	45	4.2912	23
L middle frontal gyrus	–42	12	57	4.3645	44
A + T + vs. A-T-					
B cerebellum posterior lobe	–3	–72	–27	–3.3858	11
A + T + vs. A + T-					
R middle frontal gyrus	36	36	45	–4.4847	23
L middle frontal gyrus	–33	21	57	–3.8762	28

The x, y, z coordinates is the primary peak locations in the MNI space. Cluster size > 10 voxels in ANOVA analysis, GRF corrected, voxel *p* < 0.005, cluster *p* < 0.05; Cluster size > 10 voxels in *post hoc* test, GRF corrected, voxel *p* < 0.005, cluster *p* < 0.05; A + T + , abnormal Aβ_42_ and p-tau; A + T-, abnormal Aβ_42_ and normal p-tau; A- T-, normal Aβ_42_ and p-tau; B, bilateral; L, left; R, right.

**FIGURE 2:**
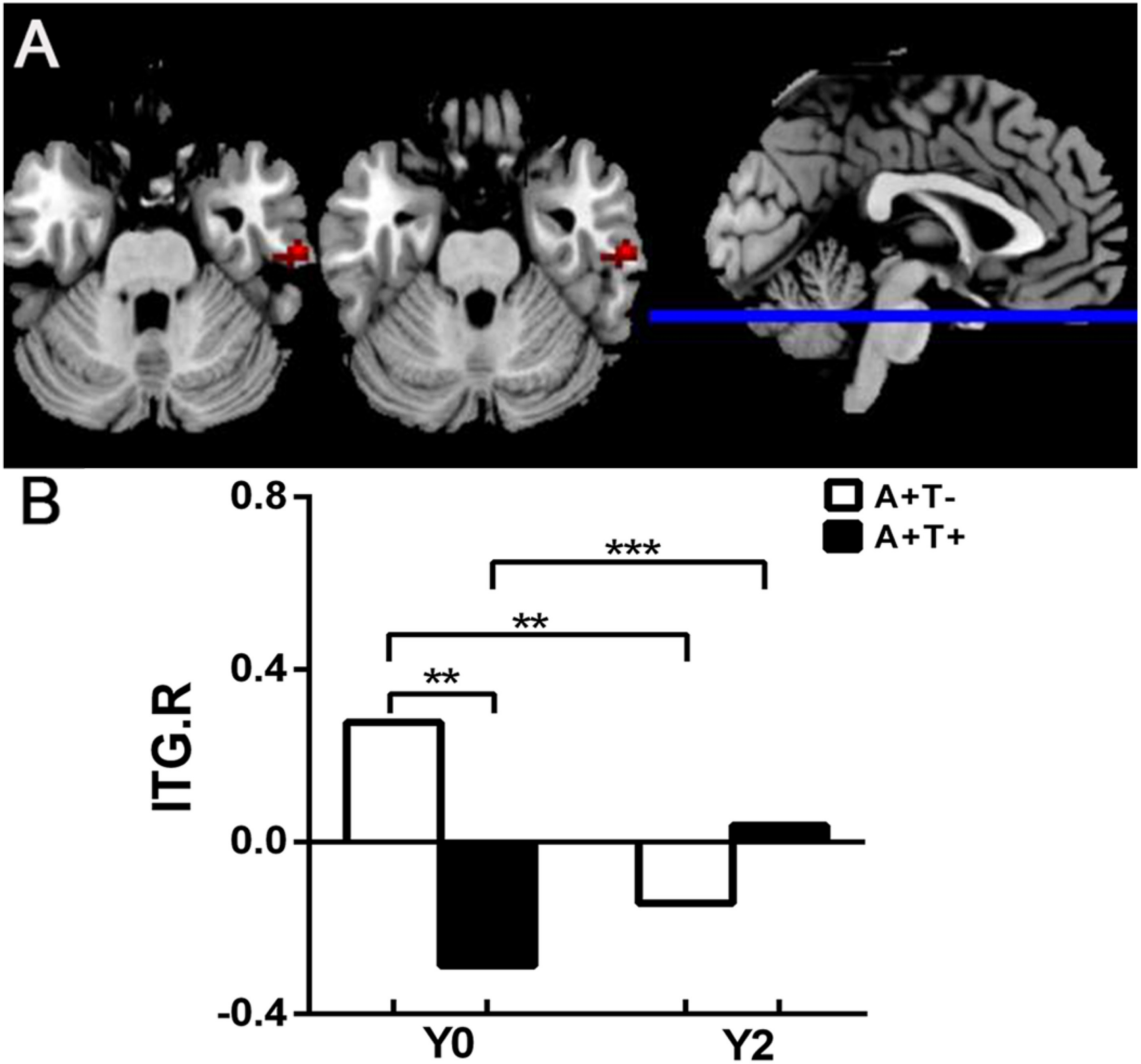
Mixed-effects model analysis of ALFF. **(A)** Brain region showing a significant Group × Time interaction in ALFF between the A + T- and A + T + groups (GRF corrected, two-sided voxel *p* < 0.005, cluster *p* < 0.05). **(B)** Mean z-standardized ALFF (zALFF) values extracted from the significant cluster, illustrating the Group × Time interaction. **p < 0.01; ***p < 0.001; A + T + , abnormal Aβ_42_ and p-tau; A + T-, abnormal Aβ_42_ and normal p-tau; A-T-; Y0, baseline time; Y2, 2-year follow-up.

The ALFF value of right inferior temporal gyrus (ITG) showed a group by time interaction: longitudinal decreased ALFF value in A + T + group, while longitudinal increased ALFF value in A + T- group. These results were obtained while accounting for age, sex, and years of education (Figure 2 and Table 3).

**TABLE 3:** Mixed-effects model analysis of ALFF in A + T- aMCI and A + T + aMCI.

Region(aal)	Peak MNI coordinate			*F/t*	Cluster number
	*x*	*y*	*z*		
R inferior temporal gyrus	60	–18	–27	23.4052	20

The x, y, z coordinates is the primary peak locations in the MNI space. GRF corrected, voxel *p* < 0.005, cluster *p* < 0.05; A + T + , abnormal Aβ_42_ and p-tau; A + T-, abnormal Aβ_42_ and normal p-tau; R, right.

### Correlation analysis

**FIGURE 3:**
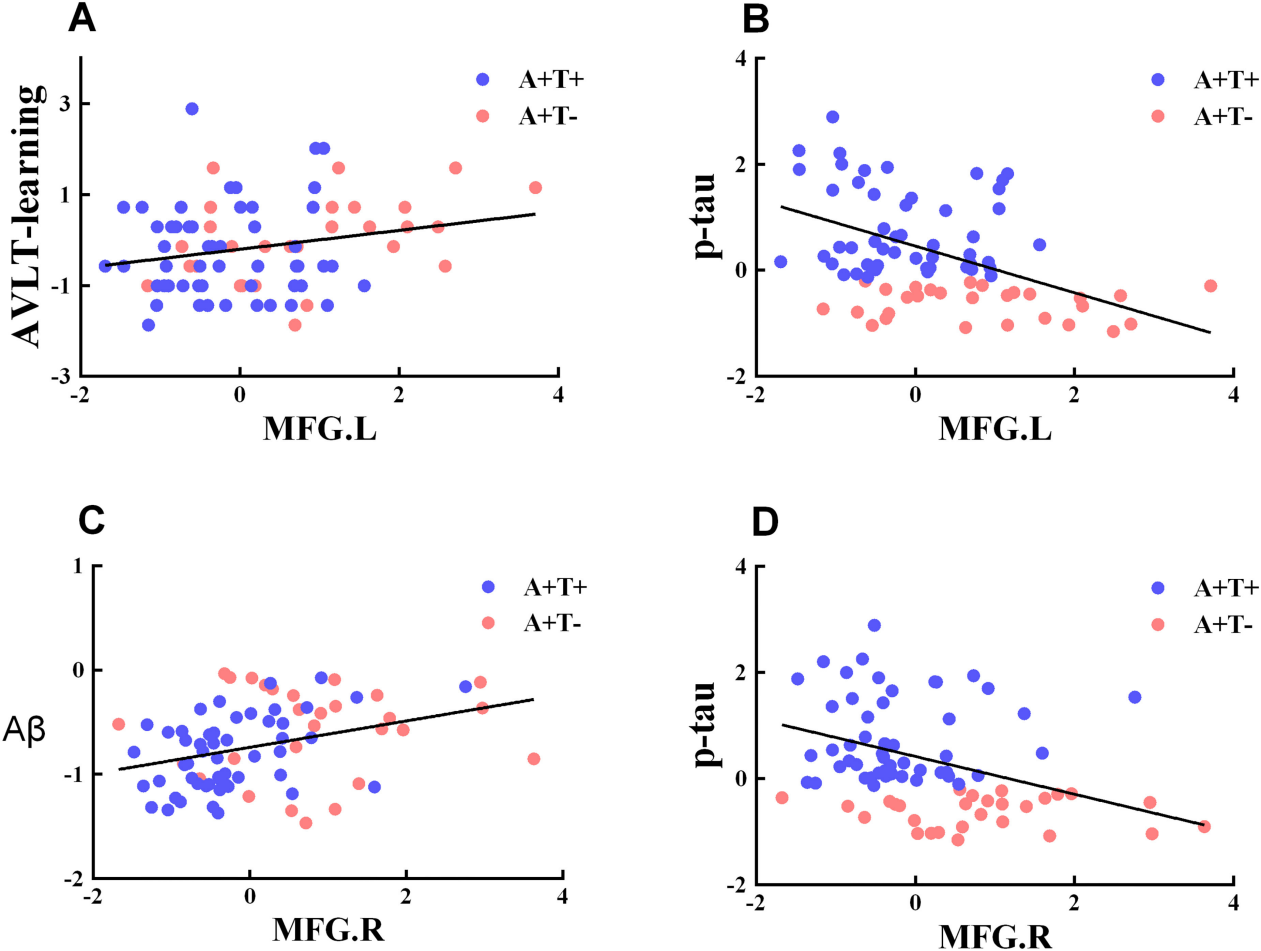
Brain regions exhibiting significant differences in ALFF and the correlation with cognitive function **(A–D)**. A + T + , abnormal Aβ_42_ and p-tau; A + T-, abnormal Aβ_42_ and normal p-tau; A- T-, normal Aβ_42_ and p-tau; MFG.L, left middle frontal gyrus; MFG.R, right middle frontal gyrus; RAVLT-learning, learning part of Rey Auditory Verbal Learning Test; Aβ, Amyloid-beta protein; p-tau, phosphorylated tau protein.

The ALFF value of the left MFG was negatively associated with p-tau (*r* = -0.428, *q* = 0.002) and positively associated with RAVLT-learning (*r* = 0.270, *q* = 0.039; Figure 3). The ALFF value of the right MFG was negatively associated with p-tau (*r* = -0.309, *q* = 0.032) and positively associated with Aβ_42_ (*r* = 0.340, *q* = 0.006). Correlation analyses were corrected for multiple comparisons using FDR (Figure 3).

## Discussion

To our knowledge, this study was the first to comprehensively examine both cross-sectional and longitudinal alterations in spontaneous brain activity among MCI patients stratified by AT biomarker profiles. During the course of our research, we made several significant discoveries: Firstly, we confirmed that cognitive impairment was indeed more severe in MCI patients with A + T + . Secondly, at the early stages of disease progression, brain function exhibited a complex pattern, with both increases and decreases occurring simultaneously; whereas in the later stages, the primary manifestation was a decline in imaging function. Furthermore, cross-sectional ALFF patterns revealed a mixed profile of functional changes: decreased activity in the right ITG, suggesting that tau pathology may accelerate regional functional deterioration over time. Lastly, there was a close correlation between changes in ALFF values and cognitive function, as well as CSF pathology proteins.

This study found that MCI with A + T + exhibited more significant cognitive impairment, and their ALFF values were lower compared to those in the A-T- and A + T- groups. Prior research indicated that the accumulation of Aβ was closely associated with fluctuations in brain activity (Jagust and Mormino, 2011; Foster et al., 2018). Researchers discovered that early Aβ deposition was related to increased brain activity in individuals with normal cognitive function and those in the early stages of MCI (Mormino et al., 2012; Schultz et al., 2017). This enhanced activity was viewed as a manifestation of maladaptive processes and excitotoxicity preceding neuronal loss (Elman et al., 2014; Corriveau-Lecavalier et al., 2020). However, as the disease progresses, these activity enhancements were subsequently followed by a reduction in brain activation, leading to a decline in cognitive abilities (Foster et al., 2018). In the latest research findings, the decline in cognitive abilities appeared to be more closely linked to tau proteins, which was consistent with the results of the present study (Hanseeuw et al., 2019; Ossenkoppele et al., 2019a; Wales and Leung, 2021). Ossenkoppele et al. found tau was more sensitive than Aβ and measures of cortical thickness for detecting early cognitive changes in preclinical AD (Ossenkoppele et al., 2019b). Moreover, recent research further suggested that the reduction in brain activity and connectivity may be more closely related to changes in tau protein levels (Ossenkoppele et al., 2019a). They proposed that tau pathology spreads through circumscribed brain networks and drives neurodegeneration (Ossenkoppele et al., 2019a). This loss of connectivity was tightly associated with the progressive structural atrophy and neuronal death induced by tau pathology, which disrupted the stability of axonal structures and interferes with axonal transport (Iqbal et al., 2005; Wales and Leung, 2021). Shigemoto et al. proposed that the coexistence of amyloid and tau in the AD-spectrum group seems to outweigh the compensatory response leading to decreased connectivity (Shigemoto et al., 2018). The results of the current study provided strong evidences for this theory. In summary, the longitudinal right ITG finding indicates a differential rate of change between biomarker-defined MCI groups, with a steeper decline in ALFF observed in the A + T + group compared to A + T-. This pattern suggests progressive functional reduction associated with greater AD pathology (Mijalkov et al., 2023). However, with only two time points, the present data support differences in slope rather than definitive non-linear trajectories.

The current study found that, compared to the A-T- group, both the A + T- and A + T + groups exhibited decreased ALFF values in the bilateral CPL. While previous research indicated that the cerebellum is primarily responsible for sensorimotor and vestibular functions, recent studies have demonstrated its significant impact on cognitive, emotional, and autonomic domains (Cutando et al., 2022). Patients with cerebellar disorders exhibited impairments in various cognitive areas and subdomains, such as verbal fluency, working memory, abstract reasoning, visual-spatial cognitive processes, social cognition, and problem-solving (Rudolph et al., 2023). The cerebellar cortex is highly folded and densely connected with prefrontal, parietal, and temporal association areas through cortico-cerebellar loops, positioning the CPL as an important hub within large-scale cognitive networks (Uwisengeyimana et al., 2020). Numerous prior articles have already revealed structural impairments and altered functional connectivity (FC) in the CPL of patients with MCI/AD, suggesting that cerebellar involvement is not merely secondary but may contribute to network-level dysfunction in the AD spectrum (Xue et al., 2024). From a clinical perspective, reduced CPL activity may reflect early disruption of cortico-cerebellar circuits supporting executive and mnemonic functions. As these circuits may be affected before overt atrophy, cerebellar functional alterations could hold potential as early imaging markers. Prior studies have suggested that cortico-cerebellar connectivity metrics may aid early detection in AD (Qi et al., 2019; Tang et al., 2021; Yao et al., 2023). Our findings further suggest CPL dysfunction may contribute to AD-related network alterations and early functional vulnerability.

Compared to the A-T- group, the A + T- group exhibited a significant enhancement in ALFF values within the bilateral MFG. As a pivotal player in memory processing, individuals with higher activity levels in the frontal cortex tend to perform better on semantic and episodic memory tasks (Zhao et al., 2014). Previous studies unveiled synaptic loss as a primary pathological hallmark of cognitive decline in AD patients, particularly prominent in the frontal cortex, inferior parietal lobe, and hippocampus (DeKosky and Scheff, 1990; Scheff et al., 2006). A research team indicated that synaptic density changes in the bilateral MFG of AD patients were closely associated with cognitive decline (Zhang et al., 2023). The increased bilateral MFG activity observed in the A + T- group may reflect a reactive compensatory response to early amyloid-related network disruption, consistent with reports of frontal hyperactivation in MCI (Grady et al., 2005). Moreover, past research also demonstrated that in cognitively intact elderly populations, individuals with higher Aβ levels but lower tau protein levels exhibited an increased FC trend within the network; conversely, when both levels are high, FC decreases (Schultz et al., 2017; Fredericks et al., 2018). Further, this study revealed a significant positive correlation between ALFF value in the MFG and Aβ_42_ levels, and a significant negative correlation with tau protein levels, which concurs with previous studies showing a negative relationship between tau deposition and FC (Sepulcre et al., 2017; Berron et al., 2020). These findings suggest that MFG functional alterations may reflect dynamic network responses to evolving AD pathology; however, the proposed compensatory mechanism remains interpretive rather than directly demonstrated.

Beyond cross-sectional group differences, our longitudinal mixed-effects model revealed a significant group-by-time interaction in the right ITG, where ALFF values declined more steeply in the A + T + group compared to the A + T- group. The ITG is known to play an essential role in semantic memory, object recognition, and higher-order visual processing (Price, 2012; Ossenkoppele et al., 2016). Previous neuroimaging studies have indicated that this region is particularly vulnerable to tau pathology during the progression of Alzheimer’s disease (Zhao et al., 2014; Sepulcre et al., 2017). The accelerated ALFF reduction in A + T + individuals may therefore reflect a tau-driven neurofunctional deterioration in the ventral visual stream. This finding supports the hypothesis that tau accumulation, especially when co-existing with amyloid pathology, leads to progressive disruption of specific cortical circuits, and may account for the worsening of visual semantic deficits in prodromal AD (Sepulcre et al., 2017; Hanseeuw et al., 2019).

Our study had some limitations. Firstly, our results indicated a significant age difference between the A + T + group and the A-T- group. Consequently, Age was included as a covariate in all analyses to account for potential confounding effects, and additional sensitivity analyses were conducted to further minimize the influence of age on the results. Nevertheless, residual confounding cannot be entirely excluded. Secondly, we subdivided MCI patients into A- T-, A + T-, and A + T + using CSF pathology proteins. However, we did not explicitly incorporate an “N” biomarker into subgroup classification. As a result, we cannot fully determine whether the observed functional alterations reflect primary amyloid/tau pathology or downstream neurodegenerative processes. In addition, CSF pathology proteins of AD pathology lack regional specificity, we cannot definitively ascertain whether our findings stem from localized or whole-brain effects of AD pathology. Future studies integrating regionally specific imaging biomarkers such as tau-PET and Aβ-PET would help clarify the spatial coupling between pathology and functional alterations. Third, global signal regression was applied during preprocessing to reduce non-neuronal noise. Although this approach is commonly used in resting-state fMRI studies, it remains methodologically debated. Therefore, the potential influence of preprocessing choices on the findings cannot be completely excluded.

## Conclusion

This study demonstrated that MCI patients with A + T + exhibited lower cognitive function and reduced spontaneous brain activity, aligning with the altered pathological mechanisms. Investigating both cross-sectional and longitudinal changes in brain function across different levels of pathological proteins provides crucial insights into the progression of AD-related pathology.

## Data Availability

Publicly available datasets were analyzed in this study. This data can be found here: the Alzheimer’s Disease Neuroimaging Initiative (ADNI) database (http://adni.loni.usc.edu).

## Footnotes

1
http://adni.loni.usc.edu

2
www.adni-info.org

3
https://ida.loni.usc.edu

4
http://rfmri.org/DPABI

5
http://www.mathworks.com/products/matlab/

## References

[B1] Berron D. van Westen D. Ossenkoppele R. Strandberg O. Hansson O. (2020). Medial temporal lobe connectivity and its associations with cognition in early Alzheimer’s disease. *Brain* 143 1233–1248. 10.1093/brain/awaa068 32252068 PMC7174043

[B2] Chen X. Lu B. Yan C. G. (2018). Reproducibility of R-fMRI metrics on the impact of different strategies for multiple comparison correction and sample sizes. *Hum. Brain Mapp.* 39 300–318. 10.1002/hbm.23843 29024299 PMC6866539

[B3] Corriveau-Lecavalier N. Duchesne S. Gauthier S. Hudon C. Kergoat M. J. Mellah S. et al. (2020). A quadratic function of activation in individuals at risk of Alzheimer’s disease. *Alzheimers Dement.* 12:e12139. 10.1002/dad2.12139 33521234 PMC7817778

[B4] Cutando L. Puighermanal E. Castell L. Tarot P. Belle M. Bertaso F. et al. (2022). Cerebellar dopamine D2 receptors regulate social behaviors. *Nat. Neurosci.* 25 900–911. 10.1038/s41593-022-01092-8 35710984

[B5] DeKosky S. T. Scheff S. W. (1990). Synapse loss in frontal cortex biopsies in Alzheimer’s disease: Correlation with cognitive severity. *Ann. Neurol.* 27 457–464. 10.1002/ana.410270502 2360787

[B6] Dubois B. Feldman H. H. Jacova C. Hampel H. Molinuevo J. L. Blennow K. et al. (2014). Advancing research diagnostic criteria for Alzheimer’s disease: The IWG-2 criteria. *Lancet Neurol.* 13 614–629. 10.1016/S1474-4422(14)70090-0 24849862

[B7] Elman J. A. Oh H. Madison C. M. Baker S. L. Vogel J. W. Marks S. M. et al. (2014). Neural compensation in older people with brain amyloid-beta deposition. *Nat. Neurosci.* 17 1316–1318. 10.1038/nn.3806 25217827 PMC4177011

[B8] Foster C. M. Kennedy K. M. Horn M. M. Hoagey D. A. Rodrigue K. M. (2018). Both hyper- and hypo-activation to cognitive challenge are associated with increased beta-amyloid deposition in healthy aging: A nonlinear effect. *Neuroimage* 166 285–292. 10.1016/j.neuroimage.2017.10.068 29108941 PMC5747976

[B9] Fredericks C. A. Sturm V. E. Brown J. A. Hua A. Y. Bilgel M. Wong D. F. et al. (2018). Early affective changes and increased connectivity in preclinical Alzheimer’s disease. *Alzheimers Dement.* 10 471–479. 10.1016/j.dadm.2018.06.002 30302368 PMC6174255

[B10] Grady C. L. McIntosh A. R. Craik F. I. (2005). Task-related activity in prefrontal cortex and its relation to recognition memory performance in young and old adults. *Neuropsychologia* 43 1466–1481. 10.1016/j.neuropsychologia.2004.12.016 15989937

[B11] Hanseeuw B. J. Lopera F. Sperling R. A. Norton D. J. Guzman-Velez E. Baena A. et al. (2019). Striatal amyloid is associated with tauopathy and memory decline in familial Alzheimer’s disease. *Alzheimers Res. Ther.* 11:17. 10.1186/s13195-019-0468-1 30717814 PMC6362587

[B12] Hansson O. Seibyl J. Stomrud E. Zetterberg H. Trojanowski J. Q. Bittner T. et al. (2018). CSF biomarkers of Alzheimer’s disease concord with amyloid-beta PET and predict clinical progression: A study of fully automated immunoassays in BioFINDER and ADNI cohorts. *Alzheimers Dement.* 14 1470–1481. 10.1016/j.jalz.2018.01.010 29499171 PMC6119541

[B13] Iqbal K. Alonso Adel C. Chen S. Chohan M. O. El-Akkad E. Gong C. X. et al. (2005). Tau pathology in Alzheimer disease and other tauopathies. *Biochim. Biophys. Acta* 1739 198–210. 10.1016/j.bbadis.2004.09.008 15615638

[B14] Jack C. R. Jr. Bennett D. A. Blennow K. Carrillo M. C. Dunn B. Haeberlein S. B. et al. (2018). NIA-AA research framework: Toward a biological definition of Alzheimer’s disease. *Alzheimers Dement.* 14 535–562. 10.1016/j.jalz.2018.02.018 29653606 PMC5958625

[B15] Jack C. R. Bennett D. A. Blennow K. Carrillo M. C. Feldman H. H. Frisoni G. B. et al. (2016). A/T/N: An unbiased descriptive classification scheme for Alzheimer disease biomarkers. *Neurology* 87 539–547. 10.1212/WNL.0000000000002923 27371494 PMC4970664

[B16] Jagust W. J. Mormino E. C. (2011). Lifespan brain activity, beta-amyloid, and Alzheimer’s disease. *Trends Cogn. Sci.* 15 520–526. 10.1016/j.tics.2011.09.004 21983147 PMC3206968

[B17] Jessen F. Wolfsgruber S. Wiese B. Bickel H. Mosch E. Kaduszkiewicz H. et al. (2014). AD dementia risk in late MCI, in early MCI, and in subjective memory impairment. *Alzheimers Dement.* 10 76–83. 10.1016/j.jalz.2012.09.017 23375567

[B18] Jia X. Z. Sun J. W. Ji G. J. Liao W. Lv Y. T. Wang J. et al. (2020). Percent amplitude of fluctuation: A simple measure for resting-state fMRI signal at single voxel level. *PLoS One* 15:e0227021. 10.1371/journal.pone.0227021 31914167 PMC6948733

[B19] Kim C. M. Montal V. Diez I. Orwig W. Sepulcre J. Alzheimer’s Disease Neuroimaging Initiative, (2021). Network interdigitations of Tau and amyloid-beta deposits define cognitive levels in aging. *Hum. Brain Mapp.* 42 2990–3004. 10.1002/hbm.25350 33955621 PMC8193537

[B20] Mijalkov M. Vereb D. Canal-Garcia A. Hinault T. Volpe G. Pereira J. B. et al. (2023). Nonlinear changes in delayed functional network topology in Alzheimer’s disease: relationship with amyloid and tau pathology. *Alzheimers Res. Ther.* 15:112. 10.1186/s13195-023-01252-3 37328909 PMC10273754

[B21] Mormino E. C. Brandel M. G. Madison C. M. Marks S. Baker S. L. Jagust W. J. (2012). Abeta Deposition in aging is associated with increases in brain activation during successful memory encoding. *Cereb. Cortex* 22 1813–1823. 10.1093/cercor/bhr255 21945849 PMC3388896

[B22] Ossenkoppele R. Iaccarino L. Schonhaut D. R. Brown J. A. La Joie R. O’Neil J. P. et al. (2019a). Tau covariance patterns in Alzheimer’s disease patients match intrinsic connectivity networks in the healthy brain. *Neuroimage Clin.* 23:101848. 10.1016/j.nicl.2019.101848 31077982 PMC6510968

[B23] Ossenkoppele R. Schonhaut D. R. Scholl M. Lockhart S. N. Ayakta N. Baker S. L. et al. (2016). Tau PET patterns mirror clinical and neuroanatomical variability in Alzheimer’s disease. *Brain* 139 1551–1567. 10.1093/brain/aww027 26962052 PMC5006248

[B24] Ossenkoppele R. Smith R. Ohlsson T. Strandberg O. Mattsson N. Insel P. S. et al. (2019b). Associations between tau, Abeta, and cortical thickness with cognition in Alzheimer disease. *Neurology* 92 e601–e612. 10.1212/WNL.0000000000006875 30626656 PMC6382060

[B25] Petersen R. C. Caracciolo B. Brayne C. Gauthier S. Jelic V. Fratiglioni L. (2014). Mild cognitive impairment: A concept in evolution. *J. Intern. Med.* 275 214–228. 10.1111/joim.12190 24605806 PMC3967548

[B26] Price C. J. (2012). A review and synthesis of the first 20 years of PET and fMRI studies of heard speech, spoken language and reading. *Neuroimage* 62 816–847. 10.1016/j.neuroimage.2012.04.062 22584224 PMC3398395

[B27] Qi Z. An Y. Zhang M. Li H. J. Lu J. (2019). Altered cerebro-cerebellar limbic network in AD spectrum: A resting-state fMRI study. *Front. Neural Circuits* 13:72. 10.3389/fncir.2019.00072 31780903 PMC6851020

[B28] Rudolph S. Badura A. Lutzu S. Pathak S. S. Thieme A. Verpeut J. L. et al. (2023). Cognitive-affective functions of the cerebellum. *J. Neurosci.* 43 7554–7564. 10.1523/JNEUROSCI.1451-23.2023 37940582 PMC10634583

[B29] Scheff S. W. Price D. A. Schmitt F. A. Mufson E. J. (2006). Hippocampal synaptic loss in early Alzheimer’s disease and mild cognitive impairment. *Neurobiol. Aging* 27 1372–1384. 10.1016/j.neurobiolaging.2005.09.012 16289476

[B30] Schultz A. P. Chhatwal J. P. Hedden T. Mormino E. C. Hanseeuw B. J. Sepulcre J. et al. (2017). Phases of hyperconnectivity and hypoconnectivity in the default mode and salience networks track with amyloid and Tau in clinically normal individuals. *J. Neurosci.* 37 4323–4331. 10.1523/JNEUROSCI.3263-16.2017 28314821 PMC5413178

[B31] Sepulcre J. Sabuncu M. R. Li Q. El Fakhri G. Sperling R. Johnson K. A. (2017). Tau and amyloid beta proteins distinctively associate to functional network changes in the aging brain. *Alzheimers Dement.* 13 1261–1269. 10.1016/j.jalz.2017.02.011 28366797 PMC5623176

[B32] Shigemoto Y. Sone D. Maikusa N. Okamura N. Furumoto S. Kudo Y. et al. (2018). Association of deposition of tau and amyloid-beta proteins with structural connectivity changes in cognitively normal older adults and Alzheimer’s disease spectrum patients. *Brain Behav.* 8:e01145. 10.1002/brb3.1145 30358161 PMC6305935

[B33] Tang F. Zhu D. Ma W. Yao Q. Li Q. Shi J. (2021). Differences changes in cerebellar functional connectivity between mild cognitive impairment and Alzheimer’s disease: A seed-based approach. *Front. Neurol.* 12:645171. 10.3389/fneur.2021.645171 34220669 PMC8248670

[B34] Tang X. Guo Z. Chen G. Sun S. Xiao S. Chen P. et al. (2024). A multimodal meta-analytical evidence of functional and structural brain abnormalities across Alzheimer’s disease spectrum. *Ageing Res. Rev.* 95:102240. 10.1016/j.arr.2024.102240 38395200

[B35] Uwisengeyimana J. D. Nguchu B. A. Wang Y. Zhang D. Liu Y. Qiu B. et al. (2020). Cognitive function and cerebellar morphometric changes relate to abnormal intra-cerebellar and cerebro-cerebellum functional connectivity in old adults. *Exp. Gerontol.* 140:111060. 10.1016/j.exger.2020.111060 32814097

[B36] Vromen E. M. de Boer S. C. M. Teunissen C. E. Rozemuller A. Sieben A. Bjerke M. et al. (2023). Biomarker A+T-: Is this Alzheimer’s disease or not? A combined CSF and pathology study. *Brain* 146 1166–1174. 10.1093/brain/awac158 35511164 PMC9976983

[B37] Wales R. M. Leung H. C. (2021). The effects of amyloid and Tau on functional network connectivity in older populations. *Brain Connect.* 11 599–612. 10.1089/brain.2020.0902 33813858

[B38] Xue C. Sun H. Hu G. Qi W. Yue Y. Rao J. et al. (2020). Disrupted patterns of rich-club and diverse-club organizations in subjective cognitive decline and amnestic mild cognitive impairment. *Front. Neurosci.* 14:575652. 10.3389/fnins.2020.575652 33177982 PMC7593791

[B39] Xue C. Sun H. Yue Y. Wang S. Qi W. Hu G. et al. (2021). Structural and functional disruption of salience network in distinguishing subjective cognitive decline and amnestic mild cognitive impairment. *ACS Chem. Neurosci.* 12 1384–1394. 10.1021/acschemneuro.1c00051 33825444

[B40] Xue C. Zheng D. Ruan Y. Guo W. Hu J. Alzheimer’s Disease Neuroimaging Initiative. (2024). Alteration in temporal-cerebellar effective connectivity can effectively distinguish stable and progressive mild cognitive impairment. *Front. Aging Neurosci.* 16:1442721. 10.3389/fnagi.2024.1442721 39267723 PMC11390694

[B41] Yao J. Song B. Shi J. Yin K. Du W. (2023). Effects of repetitive transcranial magnetic stimulation at the cerebellum on working memory. *Brain Sci.* 13 1158. 10.3390/brainsci13081158 37626514 PMC10452734

[B42] Zhang H. Wei W. Zhao M. Ma L. Jiang X. Pei H. et al. (2021). Interaction between Abeta and Tau in the pathogenesis of Alzheimer’s disease. *Int. J. Biol. Sci.* 17 2181–2192. 10.7150/ijbs.57078 34239348 PMC8241728

[B43] Zhang J. Wang J. Xu X. You Z. Huang Q. Huang Y. et al. (2023). In vivo synaptic density loss correlates with impaired functional and related structural connectivity in Alzheimer’s disease. *J. Cereb. Blood Flow Metab.* 43 977–988. 10.1177/0271678X231153730 36718002 PMC10196742

[B44] Zhao Z. Lu J. Jia X. Chao W. Han Y. Jia J. et al. (2014). Selective changes of resting-state brain oscillations in aMCI: An fMRI study using ALFF. *Biomed. Res. Int.* 2014:920902. 10.1155/2014/920902 24822220 PMC4005061

[B45] Zuo X. N. Di Martino A. Kelly C. Shehzad Z. E. Gee D. G. Klein D. F. et al. (2010). The oscillating brain: Complex and reliable. *Neuroimage* 49 1432–1445. 10.1016/j.neuroimage.2009.09.037 19782143 PMC2856476

