# Supplementary material for: Distinct patterns of spontaneous brain activity in mild cognitive impairment patients stratified by cerebrospinal fluid biomarkers
Source: Front Aging Neurosci. 2026 Mar 27;18:1724058. doi: 10.3389/fnagi.2026.1724058 (PMC13066172; doi:10.3389/fnagi.2026.1724058)
Supplement: Supplementary file 1 [file Data_Sheet_1.docx]

**Methods**

**Attrition Analysis**

Baseline demographic variables, cognitive measures, and CSF biomarkers were compared between participants who completed the 2-year follow-up and those who did not. Independent-sample t-tests were used for continuous variables, and chi-square tests were used for categorical variables.

**Sensitivity Analyses for Multi-site Effects**

To evaluate the potential influence of multi-site acquisition variability in the ADNI dataset, additional sensitivity analyses were conducted by including the site identifier as a categorical covariate in the statistical models.

For cross-sectional analyses, site was added as a covariate in the voxel-wise one-way ANOVA model. Statistical inference was performed using the same GRF correction parameters as in the primary analysis (two-sided voxel-level p < 0.005, cluster-level p < 0.05).

For longitudinal sensitivity analyses, mean zALFF values were extracted from the significant right inferior temporal gyrus (ITG) cluster identified in the voxel-wise mixed-effects model. ROI-based linear mixed-effects analyses were then performed in SPSS, with subject specified as a random effect (random intercept model). Site was modeled as a categorical fixed-effect covariate. This ROI-level analysis was conducted to verify that the longitudinal Group × Time interaction was not attributable to site-related acquisition variability.

**Sensitivity Analyses for Nonlinear Age Effects**

To evaluate whether the observed group differences in ALFF could be influenced by nonlinear age effects, additional sensitivity analyses were conducted incorporating a quadratic age term (Age²) into the statistical models. All statistical thresholds and correction procedures remained identical to those used in the primary analyses.

**Supplementary Results**

**Attrition Analysis**

No significant differences were observed in baseline age, sex distribution, or CSF biomarkers between the two groups. Participants who completed follow-up showed slightly lower education levels and modestly lower performance on selected cognitive measures (see Supplementary Table S1).

**Robustness of Findings After Site Adjustment**

The inclusion of site as a covariate did not materially alter the main cross-sectional findings. Results remained largely consistent with the primary findings, with no change in the main significant clusters. Minor variations in cluster extent were observed but did not alter the spatial pattern of results. Detailed statistics are presented in Supplementary Table S2.

The ROI-based mixed-effects model confirmed a significant Group × Time interaction in the right ITG after controlling for site effects (β = 0.747, p < 0.001). The direction and magnitude of the interaction were consistent with the primary analysis, supporting the robustness of the longitudinal finding.

**Robustness to Nonlinear Age Adjustment**

In cross-sectional analyses, inclusion of the quadratic age term (Age²) did not materially alter the spatial distribution or statistical significance of the reported ALFF group differences. The Age² term itself was not statistically significant in the regions of interest (Table S3).

In longitudinal analyses, the Group × Time interaction in the right ITG remained significant after inclusion of Age² (p < 0.05, corrected). These findings indicate that the reported ALFF alterations are unlikely to be driven by nonlinear age effects.

Table S1 Baseline characteristics of participants with and without 2-year follow-up

|  | Follow-up Completers | Non-Completers | P values (χ 2) | p values |
| --- | --- | --- | --- | --- |
| Number | 69 | 65 |  |  |
| Age (years) | 70.56(6.91) | 7179(7.31) | -1.002 | 0.318^a^ |
| Gender (F/M) | 25/44 | 28/37 | 0.656 | 0.418 |
| Years of education | 15.74(2.64) | 16.66(2.53) | -2.060 | 0.041 |
| MMSE | 27.54(2.23) | 28.12(1.67) | -1.714 | 0.089 |
| MoCA | 22.68(3.56) | 23.81(2.64) | -2.033 | 0.044 |
| RAVLT-immediate | 33.46(9.27) | 37.82(9.17) | -2.731 | 0.007 |
| RAVLT-learning | 4.01(2.25) | 4.68(2.35) | -1.669 | 0.098 |
| RAVLT-forgetting | 4.70(3.62) | 4.74(2.46) | -0.080 | 0.937 |
| RAVLT-prec-forgetting | 58.07(53.48) | 53.66(28.90) | -0.588 | 0.558 |
| LDELTOTAL | 6.41(3.21) | 7.42(3.02) | -1.871 | 0.064 |
| EM | 0.18(0.68) | 0.38(0.77) | -1.640 | 0.103 |
| EF | 0.41(0.89) | 0.40(0.97) | 0.111 | 0.912 |
| Aβ_42_ | 950.37(477.93) | 1034.71(427.00) | -1.064 | 0.289 |
| T-tau | 279.68(139.53) | 254.87(114.87) | 1.120 | 0.265 |
| P-tau | 27.79(17.35) | 14.41(13.83) | 1.243 | 0.216 |

Numbers are given as means (standard deviation, SD) unless stated otherwise. MMSE, Mini-mental State Examination; MoCA, Montreal Cognitive Assessment; RAVLT, Rey Auditory Verbal Learning Test; LDELTOTAL, Logical Memory Test; EM, episodic memory; EF, executive function; Aβ, Amyloid‐beta protein; p-tau, phosphorylated tau protein; t-tau, total tau protein.

Table S2 Baseline group differences in ALFF with site included as a covariate

| Region(aal) | Peak MNI coordinate | | | F/t | Cluster number |
| --- | --- | --- | --- | --- | --- |
|  | x | y | z |  |  |
| ANOVA | | | | | |
| B cerebellum posterior lobe | 9 | -63 | -24 | 8.475 | 60 |
| R middle frontal gyrus | 33 | 36 | 45 | 14.5313 | 39 |
| L middle frontal gyrus | -39 | 12 | 57 | 11.0132 | 54 |
| A+T- vs A-T- | | | | | |
| B cerebellum posterior lobe | 9 | -66 | -24 | -3.6595 | 44 |
| L cerebellum posterior lobe | 0 | -66 | -33 | -3.2937 | 12 |
| R middle frontal gyrus | 33 | 39 | 45 | 4.4277 | 31 |
| L middle frontal gyrus | -39 | 12 | 60 | 4.3549 | 52 |
| A+T+ vs A-T- | | | | | |
| L cerebellum posterior lobe | -3 | -72 | -27 | -3.4477 | 7 |
| A+T+ vs A+T- | | | | | |
| R middle frontal gyrus | 33 | 36 | 45 | -5.071 | 35 |
| L middle frontal gyrus | -39 | 9 | 57 | -4.1951 | 41 |

The x, y, z coordinates is the primary peak locations in the MNI space. GRF corrected, voxel p <0.005, cluster p < 0.05; A+T+, abnormal Aβ_42_ and p-tau; A+T-, abnormal Aβ_42_ and normal p-tau; A-T-, normal Aβ_42_ and p-tau; B, bilateral; L, left; R, right.

Table S3 Baseline group differences in ALFF with age^2^ included as a covariate

| Region(aal) | Peak MNI coordinate | | | F/t | Cluster number |
| --- | --- | --- | --- | --- | --- |
|  | x | y | z |  |  |
| ANOVA | | | | | |
| B cerebellum posterior lobe | 9 | -63 | -24 | 7.8753 | 45 |
| R middle frontal gyrus | 33 | 36 | 45 | 12.0101 | 30 |
| L middle frontal gyrus | -39 | 12 | 57 | 10.0566 | 41 |
| A+T- vs A-T- | | | | | |
| B cerebellum posterior lobe | 9 | -66 | -24 | -3.5895 | 42 |
| R middle frontal gyrus | 33 | 39 | 45 | 4.317 | 25 |
| L middle frontal gyrus | -42 | 12 | 57 | 4.354 | 42 |
| A+T+ vs A-T- | | | | | |
| L cerebellum posterior lobe | -3 | -72 | -27 | -3.5079 | 7 |
| A+T+ vs A+T- | | | | | |
| R middle frontal gyrus | 36 | 36 | 45 | -4.5683 | 23 |
| L middle frontal gyrus | -33 | 21 | 57 | -3.8178 | 28 |

The x, y, z coordinates is the primary peak locations in the MNI space. GRF corrected, voxel p <0.005, cluster p < 0.05; A+T+, abnormal Aβ_42_ and p-tau; A+T-, abnormal Aβ_42_ and normal p-tau; A-T-, normal Aβ_42_ and p-tau; B, bilateral; L, left; R, right.
